# Overexpression of IGF2 Alters the Transcriptional Profile of Goose Skeletal Muscle Satellite Cells

**DOI:** 10.3390/biom16040565

**Published:** 2026-04-10

**Authors:** Cui Wang, Yi Liu, Yunzhou Yang, Shufang Chen, Daqian He

**Affiliations:** 1 Institute of Animal Science and Veterinary Medicine, Shanghai Academy of Agricultural Sciences, Shanghai 201106, China; cuiwang518@saas.sh.cn (C.W.); liuyi20031194@saas.sh.cn (Y.L.); yangyunzhou@saas.sh.cn (Y.Y.); 2 NingBo Academy of Agricultural Sciences, Ningbo 315040, China; 13606780161@163.com

**Keywords:** IGF2, goose, skeletal muscle satellite cells, transcriptional profile, myogenesis

## Abstract

Insulin-like growth factor 2 (IGF2) plays a pivotal role in regulating growth and development; however, its functional involvement in skeletal muscle satellite cells (SMSCs) remains incompletely understood. To elucidate the regulatory role of IGF2, goose SMSCs were engineered to overexpress *IGF2* via lentiviral transduction, followed by comprehensive transcriptomic profiling. Comparative analysis revealed 2802 differentially expressed genes (DEGs) in *IGF2*-overexpressing cells relative to controls, comprising 1202 upregulated and 1600 downregulated genes. *IGF2* overexpression markedly activated fibrogenic programs, as evidenced by the upregulation of AP-1 complex components (*FOS*, *JUN*), extracellular matrix-related genes (*COL1A1*, *COL5A3*), and Wnt signaling receptors (*FZD1*, *FZD7*). In contrast, genes involved in myogenic differentiation and contractile function were broadly suppressed, including key myogenic transcription factors (*MEF2C*, *MEF2D*), sarcomeric structural proteins (*MYBPC1*, *ACTN2*, *MYOM3*), and metabolic enzymes. Through the construction of protein–protein interaction networks coupled with functional enrichment analysis, we observed a concerted suppression of myogenic regulatory networks critical for myofiber formation. Quantitative real-time PCR validation further confirmed the reliability of the transcriptomic data. Collectively, these findings suggest that overexpression of *IGF2* induces a phenotypic shift from myoblasts toward a fibroblast-like state, uncoupling proliferation from differentiation while enhancing fibrogenic identity. This study provides novel insights into IGF2-mediated regulatory mechanisms underlying skeletal muscle development and fibrotic processes.

## 1. Introduction

Insulin-like growth factor 2 (IGF2) is a multifunctional polypeptide hormone within the insulin-like growth factor (IGF) system [1]. Structurally similar to insulin, the mature IGF2 protein consists of 67 amino acid residues and plays a pivotal role in cellular proliferation, differentiation, and metabolism [2,3,4].

In skeletal muscle, IGF2 is a critical growth factor regulating myoblast proliferation and differentiation. In pigs, a paternally expressed quantitative trait locus at the *IGF2* gene promotes muscle growth and reduces fat deposition [5,6]. In chickens, IGF2 has been linked to growth and carcass traits [7,8]. Recent studies further reveal that IGF2 facilitates chicken myoblast differentiation by enhancing mitochondrial biogenesis via the PI3K/AKT-PGC1/NRF1/TFAM pathway and BNIP3-mediated mitophagy, thereby remodeling mitochondrial networks and improving mitochondrial function [9]. These findings extend IGF2’s function from classical growth signaling to include organelle-level metabolic reprogramming.

In geese, our previous studies have established that *IGF2* mRNA is highly expressed in leg muscle, and a genetic variant is significantly associated with body weight in Zhedong White geese (*p* < 0.05) [10]. Additional research in porcine skeletal muscle satellite cells (SMSCs) identified complex interactions between IGF2 mRNA-binding proteins (e.g., IGF2BP3) and transcription factors like SP1, impacting cell proliferation [11]. In geese, IGF2BP2 is highly expressed in muscle and, when overexpressed, alters the expression of myogenesis-related genes, including IGF1, BMPs, and FGF19 [12]. These findings suggest that the IGF2 axis in geese involves a multi-layered regulatory network, comprising both IGF2 itself and associated RNA-binding proteins.

Beyond direct IGF2 signaling, increasing evidence points to intricate post-transcriptional regulation of myogenesis via interactions among IGF2 mRNA-binding proteins and epitranscriptomic modifications. In goat SMSCs, the N6-methyladenosine (m6A) demethylase FTO promotes proliferation by stabilizing DAG1 mRNA in an IGF2BP1-dependent manner, illustrating the role of IGF2-binding proteins as m6A readers [13]. In mouse C2C12 myoblasts, IGF2BP3 functions as a novel post-transcriptional regulator of myoblast fusion through the miR-6240/Mymk axis, where it binds to and stabilizes myomaker mRNA [14]. Collectively, these studies highlight that IGF2 and its family of RNA-binding proteins cooperatively govern myogenesis through transcriptional regulation, mRNA stability, and mitochondrial dynamics.

Despite the accumulating evidence linking *IGF2* variation to growth traits, its direct functional role in goose skeletal muscle development, especially at the transcriptional level, remains poorly understood. Because SMSCs are primary stem cells responsible for postnatal myogenesis, they provide an ideal model for probing these mechanisms. However, whether *IGF2* overexpression changes the transcriptional landscape of goose SMSCs, and the identity of its downstream targets and pathways, is unknown. Here, we established an *IGF2* overexpression model in goose SMSCs, performed transcriptomic profiling, and aimed to elucidate the regulatory mechanisms by which IGF2 modulates myogenesis—thus providing theoretical guidance for improving meat production efficiency in geese.

## 2. Materials and Methods

### 2.1. Animals and Sample Collection

A total of twenty fertilized eggs from Zhedong White geese were obtained from the Wenjie Goose Breeding Department, Xiangshan Co., Ltd., China. The eggs were incubated in a standard commercial incubator (Zhonglian, China). At embryonic day 16 (E16d), embryos were harvested, and sex determination was performed via PCR amplification of the *CHD1* gene (Table 1) as previously described [15]. For tissue collection, embryos were euthanized by trained personnel using carbon dioxide inhalation followed immediately by decapitation to ensure death, as CO_2_ alone is insufficient for avian embryos. Leg muscle tissues were aseptically dissected from female embryos (n = 6) and used as the source material for SMSC isolation.

All experimental protocols involving animals were reviewed and approved by the Institutional Animal Care and Use Committee of Shanghai Academy of Agricultural Sciences (Approval No: SAASPZ0524104, approval date: 10 March 2024). This study was conducted in full compliance with the ARRIVE guidelines (https://arriveguidelines.org) and adhered to all relevant regulations governing the ethical use of animals in research.

### 2.2. Isolation and Culture of Goose SMSCs

Following sex identification, leg muscle tissues from female embryos were aseptically dissected, and residual blood vessels, adipose tissue, and connective tissue were carefully removed. The cleaned muscle samples were minced into a fine paste and subjected to enzymatic digestion at 37 °C for 50 min in high-glucose DMEM (Corning, Grand Island, NY, USA) containing 2 mg/mL Dispase II (Roche, Basel, Switzerland) and 4 mg/mL Collagenase II (Gibco, Grand Island, NY, USA). Digestion was terminated by adding an equal volume of high-glucose DMEM supplemented with 10% fetal bovine serum (FBS; Lonsera, Ciudad de la Costa, Uruguay, South America). The resulting cell suspension was passed through a 70 µm cell strainer and centrifuged at 350× *g* for 8 min at room temperature. Red blood cells were lysed using ACK lysis buffer (Gibco, USA). The cell pellet was then resuspended in DMEM/F12 medium (Gibco, USA) containing 10% FBS, 1% penicillin-streptomycin (Gibco, Grand Island, NY, USA), and 5 ng/mL basic fibroblast growth factor (bFGF; R&D Systems, Minnneapolis, MN, USA). Cells were maintained at 37 °C in a humidified incubator with 5% CO_2_ (Thermo Fisher Scientiffc, Waltham, MA, USA).

To enrich the satellite cell population, a differential plating strategy was employed to remove rapidly adhering fibroblasts and adipocytes. After 1 h of incubation, the supernatant containing non-adherent or weakly adherent cells was collected and transferred to a new culture dish. This procedure was repeated twice to further purify the SMSC population.

The purity of the isolated SMSCs was assessed by immunofluorescence staining using the myogenic marker Pax7. Briefly, cells cultured in 6-well plates were washed three times with phosphate-buffered saline (PBS) and fixed with 4% paraformaldehyde for 20 min at room temperature. After three additional washes with PBS, cells were permeabilized with 0.25% Triton X-100 in PBS for 10 min and blocked with blocking solution (2% BSA and 0.05% Triton X-100 in PBS) for 60 min at room temperature to minimize nonspecific binding. Subsequently, cells were incubated overnight at 4 °C with primary antibodies against Pax7 (Abcam, Cambridge, UK). Following three washes with PBS, cells were incubated with fluorescently labeled secondary antibodies (1:2000 dilution, Thermo Fisher, Waltham, MA, USA) for 1 h at room temperature in the dark. After washing, nuclear staining was performed using 1× DAPI solution (10 µg/mL in PBS) for 20 min in the dark. Stained cells were visualized under a fluorescence microscope (OLYMPUS, Tokyo, Japan). The enriched SMSCs were subsequently expanded in culture for use in downstream functional experiments.

### 2.3. Plasmid Construction, Lentiviral Production, and Cell Transduction

The pKLV2-gIGF2 overexpression vector was constructed by subcloning the goose IGF2 coding sequence into the pKLV2-U6gRNA5(Empty)-PGKmCherry2AGFP backbone (Addgene, Watertown, MA, USA, #67981) using NotI and EcoRI restriction sites, employing the ClonExpress II One Step Cloning Kit (Vazyme, Nanjing, China).

For lentivirus production, 293FT cells were cultured in 10 cm dishes until reaching 70–80% confluence. On the day of transfection, a DNA mixture containing 5 µg of the transfer plasmid (either pKLV2-gIGF2 or empty vector control), 4 µg of the packaging plasmid psPAX2, and 2 µg of the envelope plasmid pMD2.G was combined with 27.5 µL of liposomal transfection reagent (Yeasen, Shanghai, China) in 500 µL Opti-MEM (Gibco, Invitrogen, Waltham, MA, USA). The mixture was incubated at room temperature for 20 min to allow DNA–lipid complex formation, after which it was added dropwise to the cells. The culture medium was replaced with fresh medium 12–16 h post-transfection. Viral supernatants were harvested 72 h post-transfection, filtered through a 0.45 µm membrane, and either used immediately for transduction or stored at −80 °C.

For transduction, purified goose SMSCs were seeded in 6-well plates and cultured until reaching 80–90% confluence. The culture medium was then replaced with 1 mL of viral supernatant containing 8 µg/mL polybrene to enhance transduction efficiency. After 12–24 h of incubation, the viral supernatant was removed and replaced with fresh growth medium. At 72 h post-transduction, cells were harvested for fluorescence-activated cell sorting (FACS) to isolate transduced populations. For sorting, approximately 1 × 10^6^ cells were collected, washed with FACS buffer (PBS containing 2% FBS), and resuspended in 300 µL of the same buffer. The suspension was then passed through a 40 µm cell strainer to obtain a single-cell suspension. FACS was performed to isolate tdTomato-positive cells, yielding IGF2-overexpressing (IGF2-OE) and wild-type control (IGF2-WT) populations.

To obtain six biological replicates per group, sorted cells from six independent transduction and sorting experiments were collected separately. For each experiment, IGF2-OE and IGF2-WT cells were sorted in parallel from the same batch of transduced cells. Thus, the six samples in each group were derived from six distinct transduction sessions, ensuring biological variability. Sorted cells were immediately stored in TRIzol reagent for subsequent RNA sequencing.

### 2.4. Library Preparation and Sequencing

Total RNA was isolated from FACS-sorted IGF2-OE and IGF2-WTcells using TRIzol reagent (Invitrogen, Waltham, MA, USA) according to the manufacturer’s protocol. RNA concentration and purity were assessed using a NanoDrop NC2000 spectrophotometer (Thermo Fisher Scientific, Waltham, MA, USA), and RNA integrity was verified by agarose gel electrophoresis. Subsequently, 3 μg of high-quality total RNA per sample was used for cDNA library construction, and the remaining RNA was stored at −80 °C for downstream applications.

Sequencing libraries were prepared using the NEBNext Ultra II RNA Library Prep Kit for Illumina (New England Biolabs, Ipswich, MA, USA) following the manufacturer’s instructions. Briefly, double-stranded cDNA was purified, end-repaired, and size-selected for fragments ranging from 400 to 500 bp using AMPure XP beads (Beckman Coulter, Brea, USA). PCR amplification was performed, and the resulting products were purified again using AMPure XP beads to obtain the final library. Library quality was evaluated using an Agilent 2100 Bioanalyzer (Agilent, Santa Clara, CA, USA) with the Agilent High Sensitivity DNA Kit. The effective library concentration was determined by real-time quantitative PCR using the StepOnePlus Real-Time PCR System (Thermo Scientific, Waltham, MA, USA). High-quality libraries were submitted for sequencing at Shanghai Personal Biotechnology Co., Ltd. (Shanghai, China). Sequencing was performed on an Illumina NovaSeq 6000 platform (Illumina, San Diego, CA, USA) using a paired-end configuration.

### 2.5. Transcriptome Analysis

RNA-seq data processing and analysis were performed as previously described [16], with detailed procedures as follows. Raw sequencing reads were first processed using fastp (v0.22.0) to ensure data quality, which involved trimming 3′-end adapter sequences and removing reads with average quality scores below Q20. Cleaned reads were then aligned to the reference genome (GenBank accession No. GCF_002166845.1; NCBI) using HISAT2 (v2.1.0) [17]. Gene expression levels were quantified using HTSeq (v0.9.1) [18], and read counts were normalized using the fragments per kilobase of transcript per million mapped reads (FPKM) method [19]. Differential expression analysis was performed using DESeq2 (v1.38.3) [20], with genes exhibiting |log_2_(Fold Change)| > 1 and an adjusted *p*-value < 0.05 considered differentially expressed genes (DEGs). Bidirectional hierarchical clustering of DEGs was conducted using the pheatmap R package (v1.0.12) [21] to group genes based on similar expression patterns.

To elucidate the biological functions of DEGs, Gene Ontology (GO) enrichment analysis was performed using topGO (v2.50.0) [22], with significant enrichment defined as an adjusted *p*-value < 0.05. Kyoto Encyclopedia of Genes and Genomes (KEGG) pathway enrichment analysis was carried out using clusterProfiler (v4.6.0) [23], focusing on pathways with adjusted *p*-values < 0.05. For each identified gene cluster, enrichment maps were generated to visualize significantly enriched functional terms. Additionally, gene set enrichment analysis (GSEA) was conducted using GSEA software (v4.1.0) [24], and enrichment pathway maps were generated. Protein–protein interaction (PPI) networks of DEGs were constructed using the STRING database [25] and visualized in Cytoscape (v3.10.4) [26].

### 2.6. Validation of DEGs by qRT-PCR

To validate the RNA-seq findings, five DEGs were selected for qRT-PCR analysis. Gene-specific primers were designed using Oligo 6.0 (Table 1). Total RNA was extracted from the same biological samples used for RNA-seq. First-strand cDNA was synthesized from 1 μg of total RNA using the PrimeScript™ RT reagent Kit with gDNA Eraser (Takara, Dalian, China) according to the manufacturer’s protocol. Subsequently, qRT-PCR analysis was performed using TB Green Premix Ex Taq II on a C1000 Touch™ Thermal Cycler (Bio-Rad, Hercules, CA, USA). Relative expression levels were calculated using the 2^−∆∆Ct^ method, with β-actin serving as the internal reference gene.

### 2.7. Statistical Analyses

Data are presented as mean ± standard error of the mean (SEM). Statistical significance was determined by one-way analysis of variance (ANOVA) followed by Duncan’s post hoc test using SPSS Statistics 22.0 (SPSS Inc., Chicago, IL, USA). Significance levels were set as * *p* < 0.05, ** *p* < 0.01, and *** *p* < 0.001.

## 3. Results

### 3.1. Identification of Goose SMSCs

**Figure 1:**
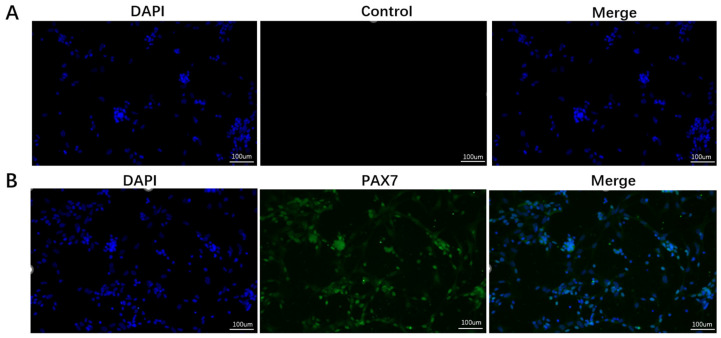
Identification of purified goose SMSCs. (**A**) Negative control with primary antibody omitted shows no specific signal. (**B**) Immunofluorescence staining confirms nuclear PAX7 expression (green) in purified SMSCs. Nuclei were counterstained with DAPI (blue). Scale bar = 100 μm.

To characterize the isolated cell population, immunofluorescence staining was performed. The cells exhibited positive staining for Pax7, a specific marker of satellite cells, whereas negative controls (with primary antibody omitted) showed no background fluorescence (Figure 1A). Co-localization of Pax7 signal within DAPI-stained nuclei confirmed the identity of the isolated cells as goose SMSCs (Figure 1B).

### 3.2. Verification of IGF2 Overexpression Efficiency in Goose SMSCs

**Figure 2:**
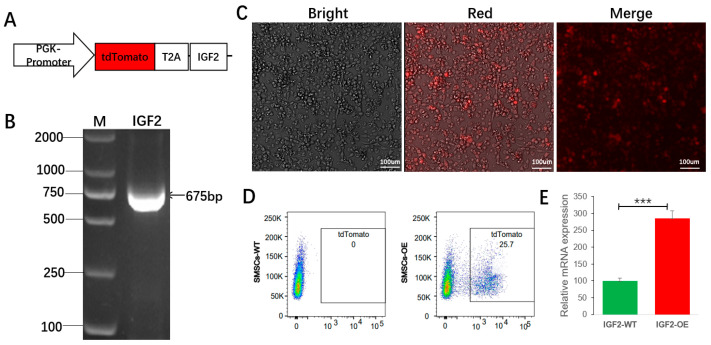
Construction and validation of IGF2 overexpression in goose SMSCs. (**A**) PCR amplification confirms the 675 bp IGF2 coding sequence. M: DNA marker. (**B**) Schematic of the overexpression construct: PGK promoter drives a tdTomato–T2A–IGF2 cassette. Original gel figures can be found at Supplementary Materials. (**C**) Bright-field, red-fluorescence and merged images showing robust tdTomato expression after 72 h post-transduction. Scale bar = 100 μm. (**D**) Flow cytometry plots showing enrichment of tdTomato-positive cells (25.7%) for RNA-seq. (**E**) qRT-PCR validation shows significantly increased IGF2 mRNA levels in the IGF2-OE group compared to IGF2-WT controls. Data are mean ± SEM. *** *p* < 0.001.

To investigate the functional role of *IGF2* in SMSCs, a lentiviral overexpression vector was constructed. The full-length goose *IGF2* coding sequence (675 bp) was cloned into the pKLV2-U6gRNA5(Empty)-PGKmCherry2AGFP backbone. As illustrated in Figure 2A, the construct employs a PGK promoter to drive expression of a tdTomato-T2A-IGF2 fusion protein, enabling real-time visualization of both transduction efficiency and *IGF2* overexpression within the same cell. Successful construction of the recombinant plasmid (pKLV2-gIGF2) was confirmed by PCR amplification, which yielded the expected 675 bp product corresponding to the goose *IGF2* coding sequence (Figure 2B).

Lentiviral particles carrying either the *IGF2* overexpression construct (IGF2-OE) or the empty vector control (IGF2-WT) were subsequently produced and used to transduce goose SMSCs. Fluorescence microscopy at 72 h post-transduction revealed efficient delivery, as evidenced by robust tdTomato expression in transduced cells (Figure 2C). To enrich the transduced population, FACS was used to isolate a distinct tdTomato-positive subset, which comprised 25.7% of the total SMSCs (Figure 2D).

To quantitatively verify the overexpression efficiency, qRT-PCR was conducted on sorted tdTomato-positive cell populations. The results demonstrated a significant increase in *IGF2* mRNA levels in IGF2-OE cells compared with IGF2-WT controls (*p* < 0.001, Figure 2E). These findings collectively confirm the successful establishment of an IGF2-overexpressing SMSC model, providing a valuable tool for subsequent gain-of-function studies.

### 3.3. Transcriptome Sequencing Reveals Widespread Transcriptional Alterations Induced by IGF2 Overexpression

**Figure 3:**
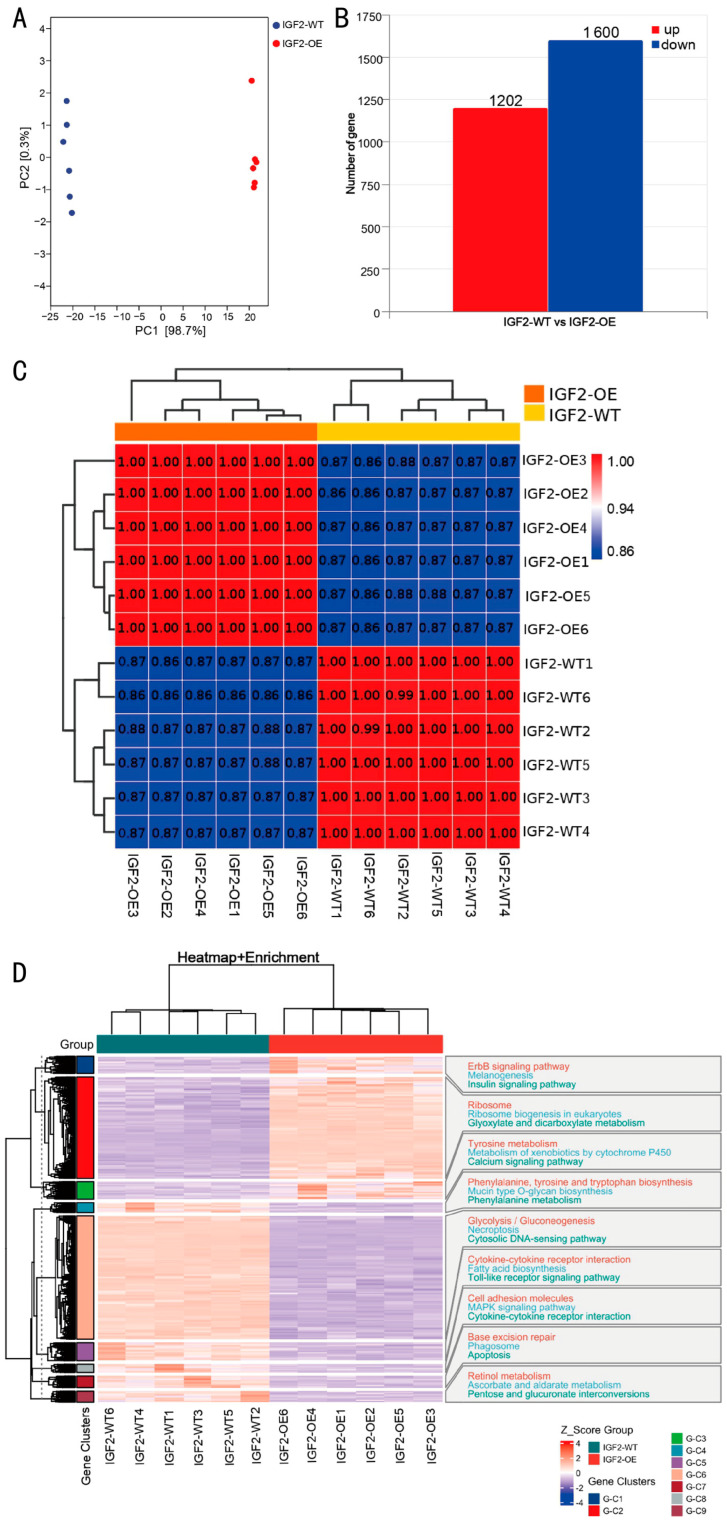
Transcriptomic profiling of goose SMSCs following *IGF2* overexpression. (**A**) Principal component analysis (PCA) of RNA-seq samples from the IGF2-OE (red) and IGF2-WT (blue) groups (n = 6 per group). The percentage of variance explained by each principal component is indicated in parentheses. (**B**) Correlation analysis of sequencing samples. (**C**) Number of DEGs between IGF2-OE and IGF2-WT groups (*p*adj < 0.05). Red and blue bars represent upregulated and downregulated genes in IGF2-OE compared to IGF2-WT, respectively. (**D**) Z-score heatmap of DEGs and their associated enriched terms.

To elucidate the transcriptomic landscape influenced by *IGF2* overexpression, RNA sequencing was performed on six IGF2-OE and six IGF2-WT goose SMSC samples. Principal component analysis (PCA) revealed clear transcriptional divergence between the two groups, with the first principal component accounting for 98.7% of the total variance, indicating that *IGF2* overexpression substantially reshaped global gene expression profiles (Figure 3A). Furthermore, Pearson correlation coefficients confirmed high intra-group consistency and distinct inter-group segregation, validating the robustness of the sequencing data (Figure 3B).

Comparative transcriptomic analysis identified a total of 2802 genes exhibiting significant differential expression between IGF2-OE and IGF2-WT cells (*p*adj < 0.05). Among these, 1202 genes were significantly upregulated (Table S1), while 1600 genes were downregulated in response to IGF2 overexpression (Figure 3C; Table S2).

To further explore the expression patterns, unsupervised hierarchical clustering was performed, which grouped the 12 samples into two primary clusters corresponding to their experimental conditions, with IGF2-OE samples (OE1–OE6) and IGF2-WT samples (WT1–WT6) forming distinct branches (Figure 3D). Examination of the expression heatmap revealed nine distinct gene clusters (G-C1 through G-C9) displaying marked differences in Z-score distributions between the two groups, highlighting coordinated transcriptional responses elicited by *IGF2* overexpression.

Functional enrichment analysis of these gene clusters revealed their involvement in a diverse array of biological processes and signaling pathways. Significantly enriched pathways, as shown in Figure 3D, included ErbB signaling, melanogenesis, insulin signaling, ribosomal function, tyrosine metabolism, calcium signaling, glycolysis/gluconeogenesis, and cytokine–cytokine receptor interactions. These pathway-level alterations point to pleiotropic effects of *IGF2* on SMSC biology, suggesting its regulatory role extends beyond classical growth-related pathways to influence metabolic, signaling, and cellular communication networks.

### 3.4. IGF2 Overexpression Activates Fibrogenic Programs While Suppressing Myogenic Differentiation

**Figure 4:**
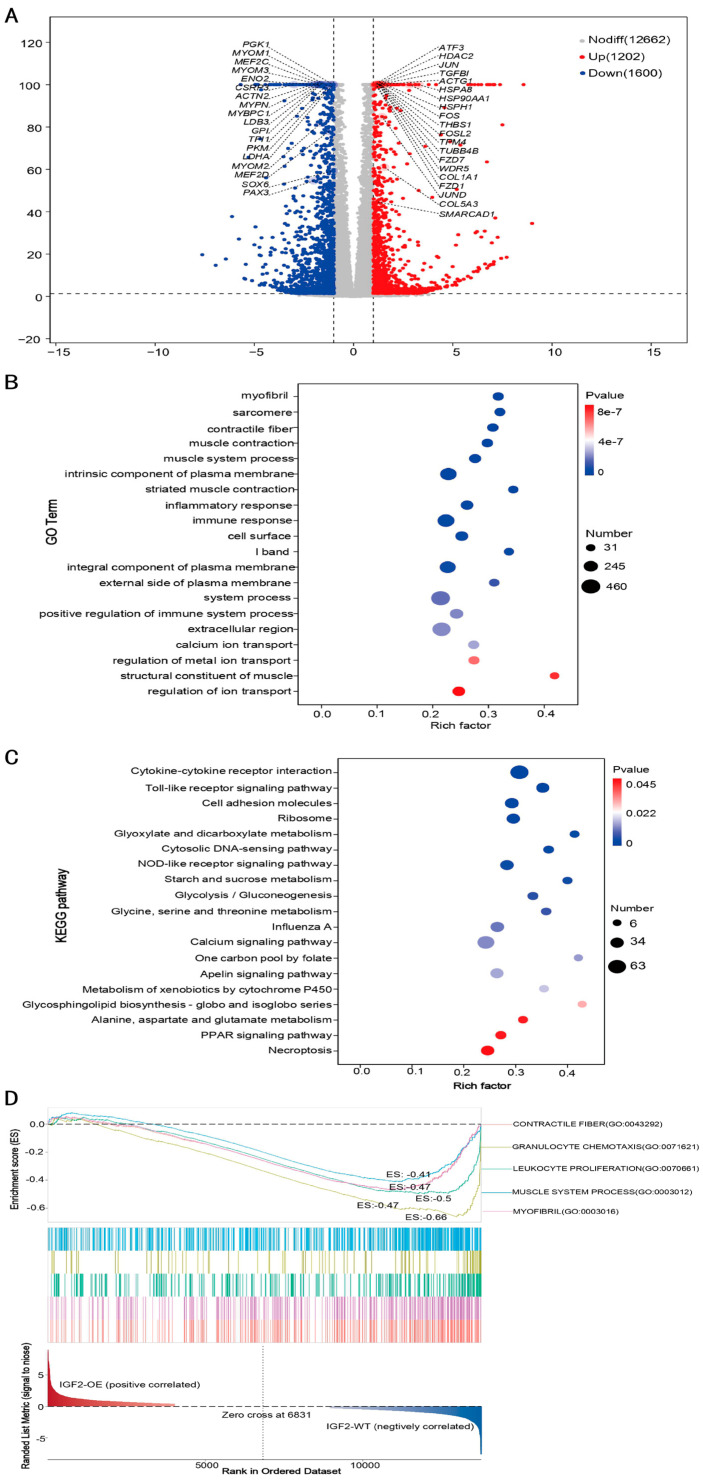
Differential gene expression analysis of goose SMSCs following IGF2 overexpression. (**A**) Volcano plot displaying DEGs between IGF2-OE and IGF2-WT groups. Red and blue dots represent significantly upregulated and downregulated genes, respectively (|log_2_FC| ≥ 1, FDR < 0.05). Gray dots indicate genes with no significant differential expression. (**B**) GO enrichment analysis of DEGs, showing the most significantly enriched biological processes, cellular components, and molecular functions. (**C**) KEGG pathway enrichment analysis of DEGs, highlighting the top significantly enriched pathways. (**D**) GSEA plots depicting the enrichment of key biological processes and signaling pathways in the IGF2-OE compared to the IGF2-WT group.

To explore the mechanistic basis by which *IGF2* overexpression dictates fate determination in goose SMSCs, we initially profiled the expression of genes related to key signaling pathways and cellular processes. The resulting heatmap uncovered marked transcriptional reprogramming, characterized by reciprocal regulation of fibrogenic and myogenic gene signatures (Figure 4A).

Specifically, IGF2 overexpression induced significant upregulation of genes associated with progenitor activation and fibrogenesis. These included AP-1 transcription factor components (*FOS*, *JUN*, *ATF3*, *JUND*, *FOSL2*), epigenetic regulators (*HDAC2*, *SMARCAD1*, *WDR5*), extracellular matrix constituents (*THBS1*, *TGFBI*, *COL1A1*, *COL5A3*), Wnt signaling receptors (*FZD1*, *FZD7*), cytoskeletal elements (*ACTG1*, *TUBB4B*, *TPM4*), and heat shock proteins (*HSP90AA1*, *HSPA8*, *HSPH1*) (Figure 4A). Conversely, a broad and coordinated downregulation was observed in genes essential for myogenic differentiation and contractile function. These included myogenic transcription factors (*MEF2C*, *MEF2D*), developmental regulators (*PAX3*, *SOX6*), and a comprehensive array of sarcomeric structural proteins, such as *MYBPC1*, *MYBPC3*, *MYOM1*, *MYOM2*, *MYOM3*, *MYPN*, *LDB3*, *ACTN2*, *CSRP3*, and *TCAP*, as well as metabolic enzymes (*LDHA*, *PKM*, *GPI*, *PGK1*, *TPI1*, *ENO2*, *SDHA*) (Figure 4A).

To validate these findings at the functional level, GO enrichment analysis of DEGs was performed. Biological processes related to muscle system development, muscle contraction, and sarcomere organization were significantly enriched among downregulated genes, consistent with the suppression of myogenic differentiation. Upregulated genes were predominantly associated with extracellular matrix organization, collagen fibril organization, and cell adhesion, thereby indicating the activation of fibrogenic programs (Figure 4B; Table S3).

KEGG pathway analysis further corroborated these observations. A marked downregulation of genes involved in cardiac muscle contraction, hypertrophic cardiomyopathy, and dilated cardiomyopathy was observed, underscoring the potential impairment of contractile function and muscle integrity. Upregulated genes, however, showed enrichment in pathways such as ECM–receptor interaction, focal adhesion, and protein processing in endoplasmic reticulum, reflecting enhanced matrix production and cellular remodeling (Figure 4C; Table S4).

Consistent with these findings, GSEA revealed significant negative enrichment of gene sets related to muscle structure and function, including contractile fiber, myofibril, and muscle system process, further confirming the widespread suppression of contractile machinery. Notably, immune-related gene sets governing granulocyte chemotaxis and leukocyte proliferation exhibited negative enrichment, supporting the notion that suppression of inflammatory pathways is coupled with the phenotypic transition (Figure 4D).

Collectively, this coordinated expression pattern demonstrates that *IGF2* overexpression drives a myoblast-to-fibroblast-like transition, uncoupling proliferation from differentiation by simultaneously activating fibrogenic programs while dismantling the myogenic differentiation machinery. These findings provide novel insights into the regulatory role of *IGF2* in skeletal muscle satellite cell fate determination.

### 3.5. PPI Network Analysis Reveals Coordinated Suppression of Myogenic Regulatory Modules

**Figure 5:**
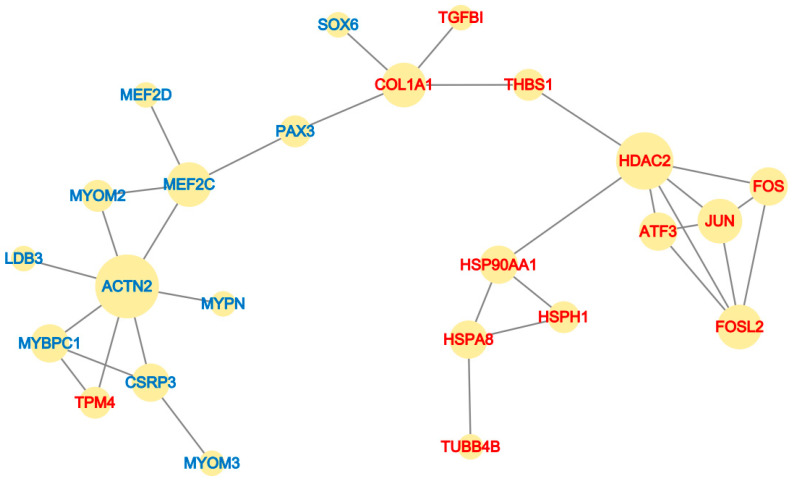
Protein–protein interaction (PPI) network of core DEGs involved in muscle development. The network was constructed using the STRING database and visualized in Cytoscape. Red nodes indicate upregulated proteins, and blue nodes indicate downregulated proteins.

Based on the STRING database, a PPI network encompassing key differentially expressed proteins was constructed and visualized in Cytoscape to systematically investigate their functional interconnections (Figure 5). The resulting interaction map revealed a clear separation between downregulated and upregulated proteins, corresponding to two distinct functional modules.

The downregulated proteins formed a densely interconnected cluster, indicating that they function together within a common regulatory module. This cluster included myogenic transcription factors (MEF2D), developmental regulators (PAX3, SOX6), and sarcomeric structural components such as ACTN2, MYPN, CSRP3, MYOM3, and MYOM2—all essential for myofibril assembly and muscle contraction.

In contrast, the upregulated proteins formed a separate cluster, comprising extracellular matrix constituents (COL1A1, TGFB1, THBS1), stress response factors (HSP90AA1, HSPA8, HSPA1), AP-1 transcription factor (FOS, JUN, FOSL2), epigenetic regulators (HDAC2), and cytoskeletal elements (TUBB4B, ATF3) (Figure 5). This cluster reflects the coordinated activation of fibrogenic and stress-related programs following *IGF2* overexpression.

The distinct modular architecture of the PPI network reinforces the notion that *IGF2* overexpression simultaneously suppresses core myogenic machinery and activates fibrogenic pathways. These findings support our transcriptomic data and suggest that *IGF2* drives a phenotypic transition from a myogenic to a fibroblast-like state at the protein interaction level.

### 3.6. Validation of RNA-Seq Data by qRT-PCR

**Figure 6:**
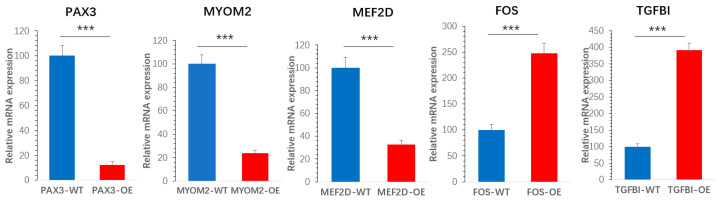
Validation of RNA-seq data by qRT-PCR. Relative mRNA expression levels of selected genes, including *PAX3*, *MYOM2*, *MEF2D*, *FOS*, and *TGFBI*, were measured in IGF2-WT and IGF2-OE goose SMSCs. Expression values were normalized to an internal control gene and are presented as mean ± SEM (n = 6 per group). Statistical significance was determined using *** *p* < 0.001.

To validate the reliability of the RNA-seq data, the expression levels of five DEGs—*PAX3*, *MYOM2*, *MEF2D*, *FOS*, and *TGFBI*—were examined in both wild-type (IGF2-WT) and IGF2-overexpressing (IGF2-OE) goose SMSCs using qRT-PCR. The qRT-PCR results showed expression trends consistent with those observed in the RNA-seq analysis. A highly significant positive correlation was found between the two datasets (*p* < 0.001, Figure 6), confirming the accuracy and reproducibility of our transcriptomic profiling.

## 4. Discussion

IGF2 is recognized as a central regulator of skeletal muscle development, promoting myoblast proliferation and differentiation. Unexpectedly, our transcriptomic analysis of goose SMSCs revealed a novel function: IGF2 overexpression induces a pronounced phenotypic shift, suppressing myogenic differentiation and concurrently activating fibrogenic programs—a process we term the “myoblast-to-fibroblast-like transition” (MFLT). This expands IGF2’s known roles and identifies it as a molecular switch that may govern the balance between muscle regeneration and pathological fibrosis.

Transcriptomic data reveal that *IGF2* overexpression significantly altered the expression of 2802 genes, indicating a dose-dependent dual role. While physiological IGF2 levels support satellite cell activation and proliferation, sustained overexpression disrupts the core myofibril assembly network, as shown by the downregulation of key myogenic regulators (e.g., *MEF2C*, *MEF2D*) and sarcomeric structural genes. Concurrent upregulation of AP-1 transcription factors (*FOS*, *JUN*, *ATF3*), extracellular matrix components (*COL1A1*, *COL5A3*, *TGFBI*), and Wnt receptors (*FZD1*, *FZD7*) suggests coordinated activation of pro-fibrotic pathways [27].

The coordinated upregulation of AP-1 family members may represent an early event in IGF2-driven fibrogenesis, given the established role of AP-1 activation in diverse tissue fibrotic processes [27,28]. The induction of *FOS*, *JUN*, and *ATF3* in our model resembles mechanisms observed in liver fibrosis, where the c-Jun/AP-1 complex promotes pathogenesis via osteopontin regulation [29]. Future studies should examine the specific composition of AP-1 dimers activated during MFLT, as distinct dimer combinations may drive different fibrogenic subprograms. Furthermore, upregulation of *TGFBI*—a known TGF-β target gene—and enrichment of ECM organization terms indicated a synergistic interaction between IGF2 and TGF-β signaling. This aligns with findings in pulmonary fibrosis, where IGF2 drives fibroblast-to-myofibroblast conversion via IGF1R/insulin receptor signaling and disrupts ECM homeostasis by upregulating TIMPs [30]. *TGFBI* upregulation implicates TGF-β signaling in IGF2-induced MFLT, potentially creating a positive feedback loop that amplifies fibrogenic signals.

Beyond AP-1 and TGF-β, upregulation of Wnt receptors *FZD1* and *FZD7* suggests Wnt signaling also contributes to MFLT. Wnt signaling alters satellite cell fate in aged muscle by promoting fibroblast differentiation [31], and the Wnt-TGF-β2 axis is involved in fibrogenic conversion in Duchenne muscular dystrophy [32]. This co-expression pattern supports the hypothesis that IGF2 promotes MFLT, at least in part, by sensitizing cells to Wnt ligands and activating a fibrogenic transcriptional program.

Notably, the transcriptional signature of IGF2-overexpressing SMSCs—particularly upregulation of ECM-related genes such as *COL1A1*, *COL5A3*, and *TGFBI*—closely resembles molecular profiles observed in human genetic myopathies. Genes involved in extracellular matrix organization are consistently dysregulated across multiple muscular dystrophies, suggesting a common pathological pathway underlying muscle degeneration [33,34]. The convergence of our IGF2-induced signature with these dystrophic patterns implies that IGF2 overexpression may recapitulate key aspects of the molecular pathology in genetic myopathies.

At the systems level, PPI analysis showed that downregulated proteins, including MEF2C, MEF2D, MYOM3, and ACTN2, form tightly interconnected modules. This coordinated suppression indicates that IGF2 overexpression systematically disrupts the myofibril assembly network rather than stochastically affecting individual genes [35,36]. Such network-level disruption provides a mechanistic basis for IGF2-mediated pathology and suggests that aberrant IGF2 activation could contribute to fibrosis in muscular dystrophies through similar regulatory perturbations [37].

While this study provides the first transcriptomic characterization of IGF2-induced MFLT, several limitations should be noted. First, conclusions are primarily based on transcriptional data; protein-level validation—especially for key regulators such as AP-1 components and TGFBI—is needed to confirm their functional involvement. Second, although the exogenous overexpression system effectively dissects IGF2 function, it may not fully recapitulate the nuanced dose-dependent effects of endogenous IGF2 or the complex microenvironment and mechanical cues present in native muscle tissue.

These considerations point to future directions. Integrating proteomic and phosphoproteomic analyses with precision gene-editing tools (e.g., CRISPR/Cas9) to establish more physiologically relevant models will enable a deeper dissection of MFLT mechanisms. In vivo studies using muscle injury or disease models will be essential to determine whether IGF2-driven MFLT contributes to fibrotic pathology and to evaluate the therapeutic potential of targeting this pathway.

## 5. Conclusions

In summary, this study demonstrates that IGF2 overexpression in goose SMSCs induces extensive transcriptomic reprogramming, resulting in a phenotypic shift from a myogenic to a fibrogenic state. The identification of 2802 differentially expressed genes indicates that IGF2 simultaneously activates fibrogenic pathways and inhibits myogenic differentiation, positioning IGF2 as a key regulator of the myoblast-to-fibroblast-like transition. These findings deepen our understanding of the molecular mechanisms linking muscle development and fibrosis, and suggest that IGF2 may serve as an important target for strategies aimed at enhancing muscle regeneration or preventing pathological fibrosis.

## Figures and Tables

**Table 1:** Primers used in this study.

Purpose	Primer Name	Primer Sequence (5′→3′)	Size (bp)	Accession Number
Sex test	CHD1-F	TGCAGAAGCAATATTACAAGT	466/326	NC_089912.1
	CHD1-R	AATTCATTATCATCTGGTGG		
Internal control	β-actin-F	TCCGTGACATCAAGGAGAAG	224	M26111.1
	β-actin-R	CATGATGGAGTTGAAGGTGG		
qRT-PCR Validation	PAX3-F	GCACTAAAATTCAACTCTCCA	204	KT380623.1
	PAX3-R	GAGAGCTGGTATGTCGGCAA		
	FOS-F	CCTGGGGCCTCCTCCTTCTAC	127	XM_066998124.1
	FOS-R	AGGTGGAGGTGTAGGTGCTG		
	MEF2D-F	TTGATGAAGAAAGCCTACGA	178	XM_066986213
	MEF2D-R	TCTCGATGATGTCCGCGTTG		
	TGFBI-F	GCGGGAAGTCAACAGTAATCA	155	XM_048057051.2
	TGFBI-R	AGACCGGTCGGAGTAGAGCTG		
	MYOM2-F	AAGGATCCGGTTTGCCAGTGA	160	XM_013177726.3
	MYOM2-R	AGCTCGACTTATTCTTTCCTCA		

## Data Availability

The datasets generated and analyzed in this study are publicly available in the NCBI BioProject database under the accession number PRJNA1433969 (https://www.ncbi.nlm.nih.gov/bioproject/PRJNA1433969), accessed on 8 March 2026.

## Supplementary Materials

The following supporting information can be downloaded at: https://www.mdpi.com/article/10.3390/biom16040565/s1, Table S1: Upregulated gene list; Table S2: Downregulated gene list; Table S3: Significantly enriched GO terms among DEGs; Table S4: KEGG pathway analysis of DEGs; Original gel figures.

## Abbreviations

The following abbreviations are used in this manuscript:

IGF2	Insulin-like growth factor 2
SMSCs	skeletal muscle satellite cells
FACS	fluorescence-activated cell sorting
DEGs	Differentially expressed genes
GSEA	gene set enrichment analysis
PCA	Principal component analysis
PPI	protein–protein interaction
MFLT	myoblast-to-fibroblast-like transition
