# Supplementary figures and images for: Overexpression of IGF2 Alters the Transcriptional Profile of Goose Skeletal Muscle Satellite Cells
Source: Biomolecules. 2026 Apr 10;16(4):565. doi: 10.3390/biom16040565 (PMC13113144; doi:10.3390/biom16040565)

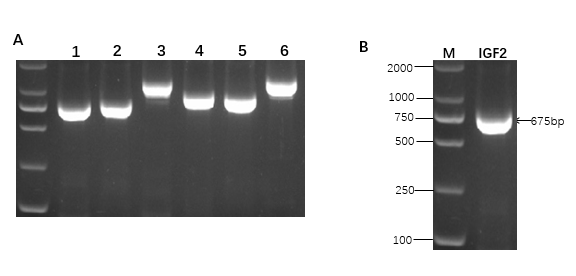

Supplement: Supplementary file 1 [file biomolecules-16-00565-s001.zip › original gel figures.png]
